# Comparative Analysis of 3D Expression Patterns of Transcription Factor Genes and Digit Fate Maps in the Developing Chick Wing

**DOI:** 10.1371/journal.pone.0018661

**Published:** 2011-04-22

**Authors:** Malcolm Fisher, Helen Downie, Monique C. M. Welten, Irene Delgado, Andrew Bain, Thorsten Planzer, Adrian Sherman, Helen Sang, Cheryll Tickle

**Affiliations:** 1 Division of Cell and Developmental Biology, University of Dundee, Dundee, Scotland, United Kingdom; 2 Department of Biology & Biochemistry, University of Bath, Claverton Down, Bath, United Kingdom; 3 The Roslin Institute and Royal (Dick) School of Veterinary Studies, The University of Edinburgh, Midlothian, Scotland, United Kingdom; Ecole Normale Supérieure de Lyon, France

## Abstract

*Hoxd13*, *Tbx2*, *Tbx3*, *Sall1* and *Sall3* genes are candidates for encoding antero-posterior positional values in the developing chick wing and specifying digit identity. In order to build up a detailed profile of gene expression patterns in cell lineages that give rise to each of the digits over time, we compared 3 dimensional (3D) expression patterns of these genes during wing development and related them to digit fate maps. 3D gene expression data at stages 21, 24 and 27 spanning early bud to digital plate formation, captured from *in situ* hybridisation whole mounts using Optical Projection Tomography (OPT) were mapped to reference wing bud models. Grafts of wing bud tissue from GFP chicken embryos were used to fate map regions of the wing bud giving rise to each digit; 3D images of the grafts were captured using OPT and mapped on to the same models. Computational analysis of the combined computerised data revealed that *Tbx2* and *Tbx3* are expressed in digit 3 and 4 progenitors at all stages, consistent with encoding stable antero-posterior positional values established in the early bud; *Hoxd13* and *Sall1* expression is more dynamic, being associated with posterior digit 3 and 4 progenitors in the early bud but later becoming associated with anterior digit 2 progenitors in the digital plate. *Sox9* expression in digit condensations lies within domains of digit progenitors defined by fate mapping; digit 3 condensations express *Hoxd13* and *Sall1*, digit 4 condensations *Hoxd13*, *Tbx3* and to a lesser extent *Tbx2*. *Sall3* is only transiently expressed in digit 3 progenitors at stage 24 together with *Sall1* and *Hoxd13*; then becomes excluded from the digital plate. These dynamic patterns of expression suggest that these genes may play different roles in digit identity either together or in combination at different stages including the digit condensation stage.

## Introduction

There are a number of developmental systems in which apparently repetitive yet discrete and distinct structures form in a particular order and position. Examples of such systems are body segments of insects and, in vertebrates, hindbrain rhombomeres, pharyngeal arches, and teeth. For all of these systems, specific sets of transcription factors have been identified which specify position and confer a particular character on what initially appear to be similar structures e.g the transcription factor odontogenic code for the mandibular primordium that leads to the different teeth arising in their appropriate positions [1]) and the transcriptional *Hox* code for rhombomere identity [reviewed 2].

The digits of the limb represent another example of repeating structures. In the chick wing, three digits with distinct morphologies, in terms of phalanx number and length, develop across the antero-posterior axis in the pattern 2, 3, and 4 (going from anterior to posterior). There has been much previous research on signals which pattern the developing chick wing, particularly on the establishment of antero-posterior polarity. It is now well-established that Sonic hedgehog (Shh) plays a pivotal role in controlling digit number and pattern [3], [4] but it is not clear which genes mediate the response to Shh signalling and encode antero-posterior positional information. There is evidence that this information is specified in the early wing bud [5], [6] but the cell condensations that give rise to the digits do not form until about 2 days later. Surprisingly digit identity is labile even at this late stage [7], [8].

The Gli proteins are the transcriptional effectors of Shh signalling and among their direct targets in the mouse limb are the 5′*Hoxd* genes. *Hoxd9-Hoxd13* are expressed in overlapping domains centred on the posterior-distal region in early mouse and chick wing buds with *Hoxd13* expression being most posteriorly and distally restricted [9], [10]. Later, as the digital plate develops, *Hoxd13* is expressed throughout whereas the other 5′ *Hox* genes are expressed more posteriorly [11] such that anterior cells express only *Hoxd13*. It has been suggested that this *Hoxd13* expression in anterior digital plate is not only a signature for digit 1 [12] but controls its identity [13]. When *Hoxd11*, for example, is over-expressed throughout chick wing buds so that the anterior domain of specific *Hoxd13* expression is abolished, an additional digit 2 forms in the wing and the anterior digit in the leg was posteriorized [14], although there are also other interpretations for these pattern changes.

Other candidate genes involved in specifying antero-posterior positional values and digit identity are suggested by the striking parallels between antero-posterior patterning of chick wing digits and of fly wing venation. In the fly wing, Hedgehog signalling determines the number and pattern of veins and induces expression of *Dpp*. As a result of Hh and Dpp signalling, each vein expresses a particular combination of genes encoding transcription factors, including optomotor blind (omb), a T-box family member and the Spalt zinc finger transcription factor, a downstream transcriptional target of the Dpp signalling pathway [15], [16]. In the chick wing bud, Shh induces expression of *Bmp2*, a *Dpp* relative [17], [18]. Homologs of the *omb* gene, the vertebrate Tbox genes *Tbx2* and *Tbx3* [19] and the *Spalt* homologues, *Sall1* [20] and *Sall3* [21] are also expressed in chick wing buds. Expression of these genes in both chick wings and mouse limb buds is regulated by the Shh/Bmp signalling cascade [22], although *Tbx2* and *Tbx3* may also be upstream of *Shh* [23], [24]. Recent analyses of transcriptional profiles of anterior versus posterior regions of both mouse limb and chick wing buds identified both *Bmp2* and *Sall1* as “posterior” genes [25], [26] and there are Gli binding sites upstream of these mouse genes [25].

There are tantalising clues that *Tbx* and *Spalt* genes might contribute to a transcriptional code for digit identity. There is one report that over-expression of either *Tbx3* or *Tbx2* in chick leg buds leads to toes becoming more posterior in character [27] while in human patients with mammary ulnar syndrome, associated with haplo-insufficiency of *Tbx3*, posterior digits can be lost or abnormally spaced [28]. In fore-limbs of *Sall1* and *Sall3* double knock-out mouse embryos, the anterior digits and most of the carpal bones are lost [29] while Townes-Brocks syndrome, in which truncated forms of *Sall1* are produced [30] is characterised by thumb malformations, mostly triphalangeal thumbs, which might be considered to represent a change in digit identity.

It is possible that different combinations of transcription factors contribute to specifying antero-posterior positional values and digit identity at different stages. Therefore it is important to document the combinations of transcription factors expressed in cells that will form the different digits during development. Here we systematically compared 3D expression patterns of *Hoxd13*, *Sall1*, *Sall3*, *Tbx2*, *Tbx3* at three different stages of chick wing development, 21, 25 and 27 Hamburger- Hamilton stages [31], using Optical Projection Tomography microscopy [32]. At stage 21, about 12 hours after the onset of *Shh* expression in the wing bud, all three digits appear to have been specified [5]; stages 25 and 27 are about 24 and 48 hours later respectively. At stage 27, digit cartilage condensations expressing *Sox9* have begun to form.

Previous fate maps of the chick wing using DiI labelling to trace cell lineages showed that all three digits come from cells in the posterior part of the early wing bud tip and that this region expands across the antero-posterior axis as the bud grows out [33], [6]. Here we made long term fate maps of the regions of the early chick wing bud that give rise to each of the three wing digits by grafting wing bud tissue from embryos of the transgenic GFP chicken line [34]. We then mapped both gene expression patterns and fate maps onto the same reference wing bud models and, using computational methods, determined the transcription factor genes expressed by the cells that give rise to the different digits.

## Results

### Gene expression patterns in 3D

#### 3D gene expression patterns at stage 21

**Figure 1:**
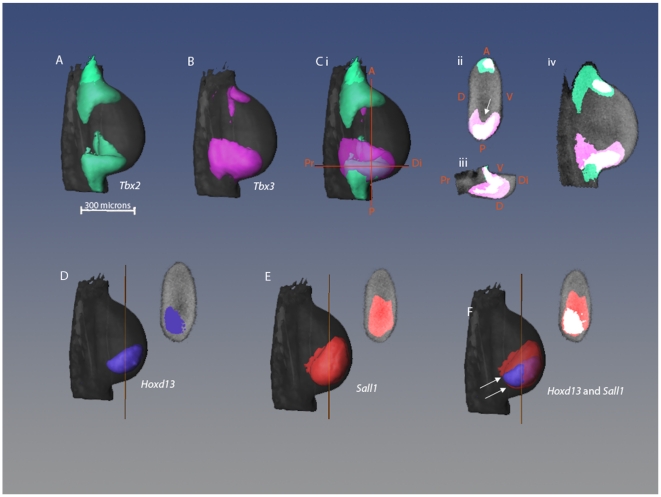
Comparison of 3D gene expression patterns in stage 21 wing bud. (A–C) Dorsal views of 3D isosurface representations of a stage 21 wing bud with expression patterns of *Tbx2* (A), *Tbx3* (B) and both *Tbx2* and *Tbx3* shown together (C). Orange lines represent the positions of the sections shown in Cii and Ciii. (Cii–Civ) 2D virtual sections of limb bud showing *Tbx2* and *Tbx3* expression where overlapping regions are shown in white, *Tbx2* alone in green and *Tbx3* alone in pink. (Civ) Sagittal section through middle of limb. A = anterior, P = posterior, D = dorsal, V = ventral, Pr = proximal, Di = distal. (D–F) Dorsal views of same wing bud showing expression patterns of *Hoxd13* (D), *Sall1* (E), and *Hoxd13* and S*all1* together with the overlapping region in white (F). Sections are shown in the same orientation as Cii, and the orange lines indicate the section position along the proximo-distal axis.

*Tbx2* and *Tbx3* expression patterns are very similar with two distinct stripes: one running along the anterior margin of the wing bud, the other along the posterior margin [19], [36], [37]. 3D analysis (Figure 1, A–C) shows that in the posterior margin, *Tbx2* expression occurs entirely within the expression domain of *Tbx3* (Figure 1, C ii–iv) while, in the anterior margin, expression of *Tbx3* is entirely overlapped by *Tbx2* expression. Posterior *Tbx3* expression is not uniform across the dorso-ventral axis, but instead forms a cup shape (indicated by white arrow, Figure 1, C.ii) whereas *Tbx2* expression is uniform. *Hoxd13* is expressed in the posterior-distal region of the wing bud (Figure 1D and F) and dorsally skewed (Figure 1, D) as previously described [38]. *Sall1* is expressed in a single posterior and distal domain which is uniform across the dorso-ventral axis (Figure 1, E). At this stage, there is no *Sall3* expression. Comparison of *Sall1* gene expression with that of *Hoxd13* shows that the *Hoxd13* expression domain almost entirely falls within the *Sall1* domain (white arrows, 1, F).

#### 3D gene expression patterns at stage 24

**Figure 2:**
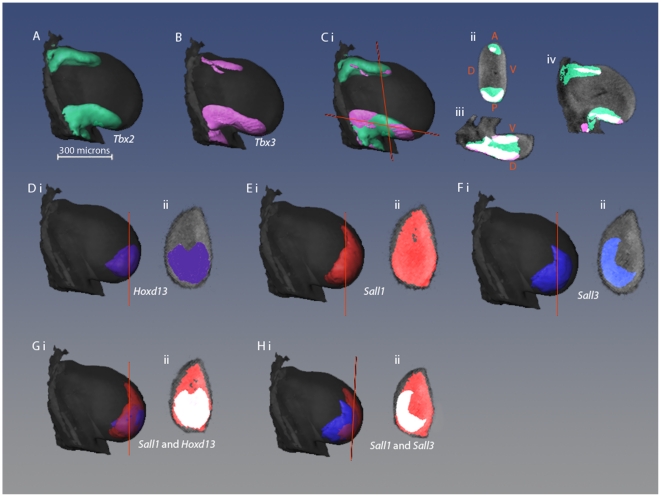
Comparison of 3D gene expression patterns in stage 24 wing bud. (A–C) Dorsal views of 3D isosurface representations of a stage 24 wing bud with expression patterns of *Tbx2* (A), *Tbx3* (B) and both *Tbx2* and *Tbx3* shown together (C). Orange lines represent the positions of the sections shown in Cii and Ciii. (Cii–Civ) 2D virtual sections of limb bud showing *Tbx2* and *Tbx3* expression where overlapping regions are shown in white, *Tbx2* alone in green and *Tbx3* alone in pink. (D–F) Dorsal views (i) and virtual sections (ii) of a limb bud showing expression patterns of *Hoxd13* (D), *Sall1* (E), and *Sall3* (F). (G&H) Pair-wise comparisons of *Sall1* and *Hoxd13* (G) and *Sall1* and *Sall3* (H) as dorsal views (i) and as virtual sections where overlapping regions are shown in white (ii).

*Tbx2* and *Tbx3* are shown separately and overlapping (Figure 2, A–C). By this stage, the anterior stripes do not extend as far distally as the posterior stripes and are also thinner [36]. The overlap in *Tbx3* and *Tbx2* expression domains is shown in sections, in Figure 2, C ii–iv. In both anterior and posterior stripes, *Tbx3* expression is almost entirely overlapped by that of *Tbx2* (71% of *Tbx3* expression occurs within the same domain as *Tbx2* expression) although there are also regions of the limb bud expressing *Tbx2* alone. As at the earlier stage, there are differences in the extent of expression along the dorso-ventral axis with *Tbx3* expression again being cup-shaped (Figure 2 Cii, iii).

*Hoxd13* expression is also posterior and distal as at stage 21 but no longer dorsally skewed (Figure 2 D i,ii,).

*Sall1* transcripts are found across the posterior and distal parts of the limb. Sections through the distal region of the limb show that the expression spreads almost completely across the A-P axis (Figure 2, E.ii). *Sall3* is also expressed by this stage, in a posterior-distal region of the bud ( Figure 2Fi) but, unlike *Sall1*, expression is dorsally skewed and the domain has a curved shape in sections(Figure 2 F ii, see comparison Figure 2 H i,ii). Thus there is an anterior ventral domain in the wing bud which expresses just *Sall1* although 72% of the *Sall1*- expressing region also expresses *Sall3*.

*Sall1* reaches further towards the anterior of the limb than *Hoxd13* (Figure 2 G I, ii) but 84% of the total volume of *Hoxd13* expression overlaps with *Sall1* expression.

#### 3D gene expression patterns at stage 27

**Figure 3:**
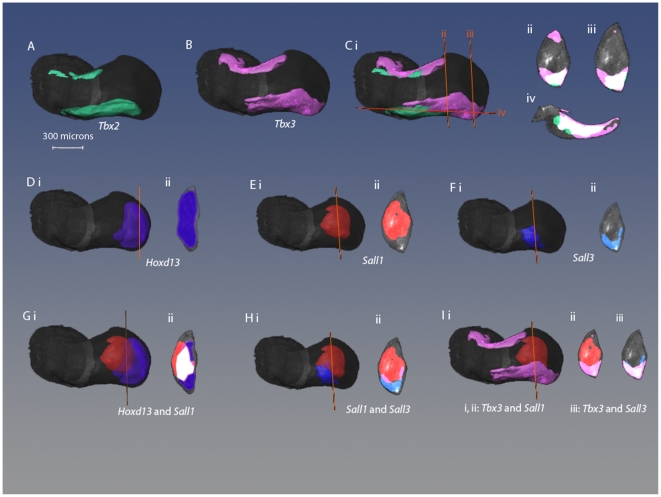
Comparison of 3D gene expression patterns in stage 27 wing bud. (A–C) Expression patterns of *Tbx2* (A) and *Tbx3* (B) and both patterns together (C). (Cii–iv) Virtual sections of Ci where the positions of the sections are indicated by corresponding orange lines. Sections are shown in same orientation as in Figures 3.1 and 3.3, although sections are shown at 2 positions along the proximo-distal axis. (D–F) *Hoxd13*, *Sall1*, and *Sall3* expression patterns shown as dorsal views (i) and as virtual sections (ii). (G–I) Comparisons of *Sall1* and *Hoxd13*, *Sall1* and *Sall3*,*Tbx3* and *Sall1*, *Tbx3* and *Sall3*, respectively. Shown as dorsal views and as virtual sections.

*Tbx2* and *Tbx3* are still expressed in anterior and posterior stripes with *Tbx2* stripes not extending as far distally as *Tbx3* and also being thinner (Figure 3A,B,C ). As at the earlier stages, the shape of the *Tbx3* domain across the dorso-ventral axis appears cup-shaped although at this stage *Tbx3* appears slightly skewed ventrally (Figure 3C ii,iii,iv). Overall 64% of *Tbx2* expression shares its domain of expression with *Tbx3* but this represents 45% of the total volume of *Tbx3* expression. So, in fact, a substantial amount of *Tbx3* expression at this stage occurs outside the domain of *Tbx2*.

At this stage *Sall1* expression has shifted away from the distal tip of the limb bud and *Sall1* is not expressed at either the anterior or posterior margins of the wing bud (Figure 3 Ei,ii) . Along the dorso-ventral axis, expression seems to be skewed towards the dorsal side. In contrast to the previous stage studied, expression of *Sall3* is now quite different to that of *Sall1* and now is almost complementary. Although expression is seen at the same proximo-distal level of the wing bud, *Sall3* is only expressed at the posterior margin of the wing bud and expression is markedly skewed dorsally (Figure 3 Fi,ii). Comparing these expression patterns in section, *Sall1* expression almost completely nestles in the cup shape of *Sall3* expression (Figure 3 H,iii).

*Hoxd13* continues to be expressed distally but extends more anteriorly and viewed in section, appears to be almost throughout the tip of the wing bud (Figure 3 Di,ii). Compared with earlier stages in development, only 16% of *Hoxd13* expression overlaps with that of *Sall1*. We also made pair-wise comparisons between *Sall1* and *Tbx3* expression (Figure 3 l i, ii) and *Sall3* and *Tbx3* (Figure 3 I iii). It is striking the way in which *Sall1* expression sits neatly inside the cup shape of *Tbx3* expression with no overlap (Figure 3 l ii). The posterior stripe of *Tbx3* expression and *Sall3* expression at this stage seem to share the same cup-shaped domain with respect to the dorso-ventral axis and 73% of *Sall3* expression is overlapped by the posterior stripe of *Tbx3* (Figure 3 l iii).

### Early skeletal development

In order to compare the gene expression patterns in stage 27 chick wing buds with the positions in which the skeletal elements develop, we stained wing buds with Alcian green to show cartilage and also carried out *in situ* hybridisation for *Sox9* expression to reveal condensations of precartilage cells [38].

**Figure 4:**
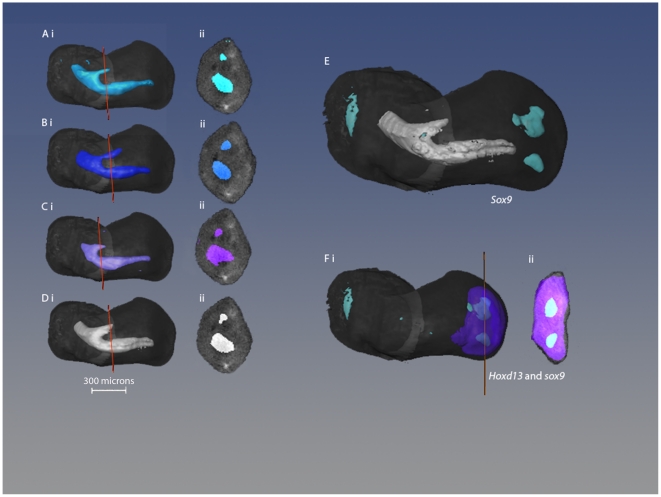
Early skeletal formation in the stage 27 wing bud. (A–D) 3D pattern of cartilage formation in the stage 27. (A–C) Isosurface representations of OPT scans of stage 27 chick wing buds stained with alcian green and displayed in dorsal views (i) and in virtual sections (ii) with section positions marked by orange lines. (D) Median of scans A–C, indicated by white box. (E) Median pattern of cartilage (light grey) together with median *Sox9* expression. (F) Median *Sox9* expression together with *Hoxd13* expression as dorsal view (i) and virtual section (ii) where all *Sox9* expression is overlapped by *Hoxd13* expression.

Figure 4 (A–C) shows individual scans of three Alcian green stained wings that contributed to the median model (Figure 4 D). The slight differences in individual scans reflect the dynamic nature of the process of cartilage differentiation. However, the median of these scans (Figure 4, D) still provides a reliable representation of the pattern of cartilage differentiation at this stage and pre-figures humerus, radius and ulna. In contrast, *Sox9* is expressed in two distinct condensations of cells (Figure 4, E; see previous description [39]) which will give rise to digits 3 and 4. Pair-wise comparison of expression patterns of *Hoxd13* and *Sox9* shows, as would be expected, that the condensations for digits 3 and 4 fall within the *Hoxd13* domain of expression and furthermore that the condensing cells express *Hoxd13*.

### Fate mapping using grafts from GFP chick embryos

**Figure 5:**
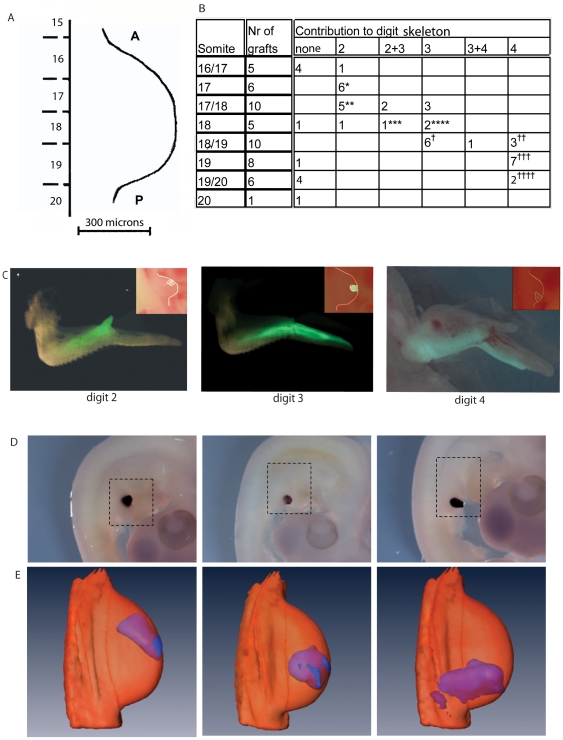
Fate maps of stage 21 chick wing buds and their digitization. (A–C) Fate maps of stage 21 chick wing buds made by replacing tissue at different positions around the antero-posterior margin with equivalent pieces of tissue from stage 21 wing buds from GFP chick embryos, whole mount *in situ* hybridisation with GFP probes, digitized and mapped to reference models. (A) Somite position and number in relation to the wing bud used to record position of grafts. A = anterior P = posterior. (B) Table summarizing contributions of grafts to individual digits. * 1 truncated, 1 in dig2+thin stripe dig3; ** 1 dig2+thin stripe dig3, 1 malformed dig2; *** 1 dig2+prox.dig3; ****1 dig3+thin stripe dig4; † 1 dig3 truncated, 1 dig3; dig4 absent, 1 dig3; dig4 separate; †† 2 slightly separate; †††1 dig4 and prox. dig3; separate dig.4, 1 dig4 slightly separated; †††† 1 post.half of dig3, separate dig4, 1 dig4 slightly separated. (C) Images showing examples of grafts ( inset) and their subsequent fate in 10 day wings, graft opposite somite 17 giving rise to digit 2 ( left panel), graft opposite somite 18 giving rise to digit 3 ( middle panel), graft opposite somite 19 giving rise to digit 4 ( right panel). (D) Whole mount *in situ* hybridisation for GFP on embryos 4 hours after GFP-expressing cells were grafted into chick wing buds in positions where they would give rise to digit 2, 3 and 4. (E) 3D mapping of *in situ* hybridisation data captured by OPT on to the stage 21 reference wing bud and visualised with Amira software.

**Figure 6:**
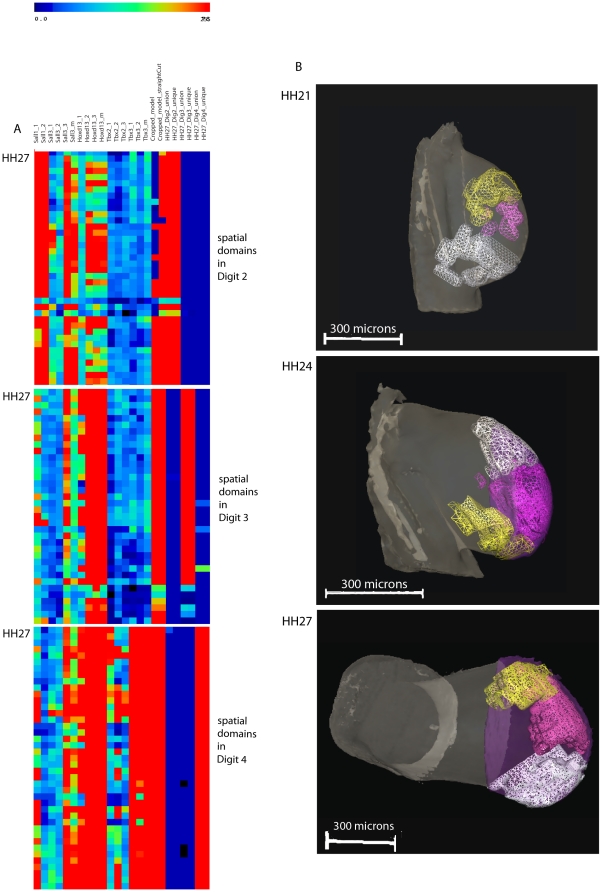
Heat maps of fate maps and gene expression patterns and 3D representations of the unique spatial domains for each digit at stage 21, 24 and 27. (A) Selection of the 2D matrix heat map showing gene expression data occurring only in both the unique digit domains and cropped domain in stage 27 wing bud. Spatial domains for each digit are non-overlapping. Red indicates high expression levels for the transcription factor genes and high signal for the GFP probe and blue: low expression levels. (B) Unique spatial domain clusters for each digit at stage 21, 24 and 27 derived from *in situ* hybridisation of GFP-expressing grafts shown as meshes. At stages 24 and 27, only the distal regions of the fate maps were used. Yellow: digit 2; magenta: digit 3; white: digit 4; violet: straight cropped domain. Note that in the view of the stage 24 wing bud, the cropped domain is hidden by the digit domains. Note that in the view of the stage 27 wing bud shown, the unique domains for digit 2 and digit 3 seem to be overlapping, but this is not the case as can be seen in Movie S3.

In order to create long-term fate maps of digit progenitors, regions of the margin of stage 21 wing buds were replaced with equivalent regions from wing buds of GFP transgenic chickens. Grafts were placed at different antero-posterior levels in wing buds, using somites and somite boundaries as reference points [33 (Figure 5A,C insets)] and the subsequent contribution to the digits was assessed in 10 day wings ( Figure 5C). The results are summarised in Figure 5, panel B. Grafts made opposite somite 17 contributed to digit 2, grafts opposite somite 18 or 18/19 mostly contributed to digit 3 and grafts opposite somite 19 to digit 4. Grafts made opposite somite 19/20 mostly formed just a thin stripe along the posterior border of digit 4. The fate maps made here are consistent with previous fate maps made with DiI and with quail grafts [33], [40]. In order to visualise these data in 3D at stages 21, 24 and 27, GFP expressing grafts were performed opposite somite 17, opposite somite 18 and opposite somite 19 to mark progenitors of digit2, digit 3 and digit 4 respectively and grafted wings were fixed at 4, 24 and 48 hours. Whole mount *in situ* hybridisation was then carried using a GFP probe (Figure 6D) and GFP expression visualised in 3D using OPT (Figure 6E).

### Computational analysis of gene expression patterns in relation to cell fate

In order to compare gene expression patterns and fate maps, all the data were accumulated onto stage 21, stage 24 and stage 27 reference wing models. For each gene, 3 replicates were mapped and a median derived. For the GFP grafts, 3 replicates were mapped for each digit, where possible and a median again derived. We then divided each reference wing bud into spatial domains of 5×5×5 voxels as previously described [26], [37] with 945 spatial domains at stage 21, 2072 at stage 24 and 4444 at stage 27) and calculated mean signal intensity of expression of all the genes, including the *GFP* gene in each spatial domain. These data were then automatically tabulated in a tab delimited file to generate a matrix of gene expression patterns across all spatial domains. Inspection of the matrices showed that, because grafts were not all exactly the same size, some spatial domains contained GFP expression from the fate maps of adjacent digits. Therefore we identified for each digit at all three stages, both unique sets of spatial domains containing non-overlapping GFP expression and union sets of spatial domains incorporating the spatial domains containing *GFP*-expression from all the grafts for that digit (cf Experimental procedures). As the grafts made at stage 21 give rise not only to digits but also to more proximal structures (Figure 5C), we also “cropped” GFP-expressing sets of spatial domains at stages 24 and 27 (see methods) so that only the distal part of the domains was considered.

To analyse the genes that were expressed in each unique digit domain, we applied a hierarchical clustering method (Pearson Correlation) to the gene expression data and the spatial domains to cluster spatial domains of gene expression. Using the tab delimited file in which the data were automatically organised after computational analysis, we selected the spatial domains that contained both the unique domain for each digit and the cropped domains. The spatial domains representing each digit were imported into Amira to produce 3D visualisations. Although *in situ* hybridisation patterns for most genes did not show any ‘noise’, we found a weak ubiquitous background signal in the OPT scans for some genes (especially *Tbx2*). Signal intensity readout of the OPT machine is given in an 8-bits greyscale, from 0–255 scales of grey. Using the Amira Voltex feature, we determined that the threshold at which we no longer observed background signal was 50 on the 8 bits greyscale. This threshold was then used for all gene expression patterns. The tab delimited files were then processed with Excel to calculate the percentage of spatial domains in which expression of a gene is >50 on an 8 – bit greyscale.

Figure 6A shows subsets of the hierarchically clustered 2D matrix representing spatial domains for stage 27. Note that this is a representation of the spatial domain clustering but not the gene clustering. The rows in Figure 6A correspond to particular spatial domains and the columns represent expression of the different genes or GFP- expressing digit progenitors in each spatial domain. Replicates of individual expression patterns cluster together (data not shown) as do union and unique domains for each digit. The three matrix subsets represent the spatial domains in each of the digit domains. Thus, for example, in the upper subset, GFP expression in grafts that give rise to digit 2 ( the unique digit 2 domain) is present in almost all the spatial domains (red column) but there is no GFP expression associated with grafts that give rise to digit 3 or digit 4 ( blue columns; unique digit3 and digit 4 domains). Figure 6B shows a 3D visualisation of the unique GFP expressing domains that give rise to each of the 3 digits at the 3 different stages. Movies of these domains are provided in the supplementary movies S1, S2 and S3.

**Figure 7:**
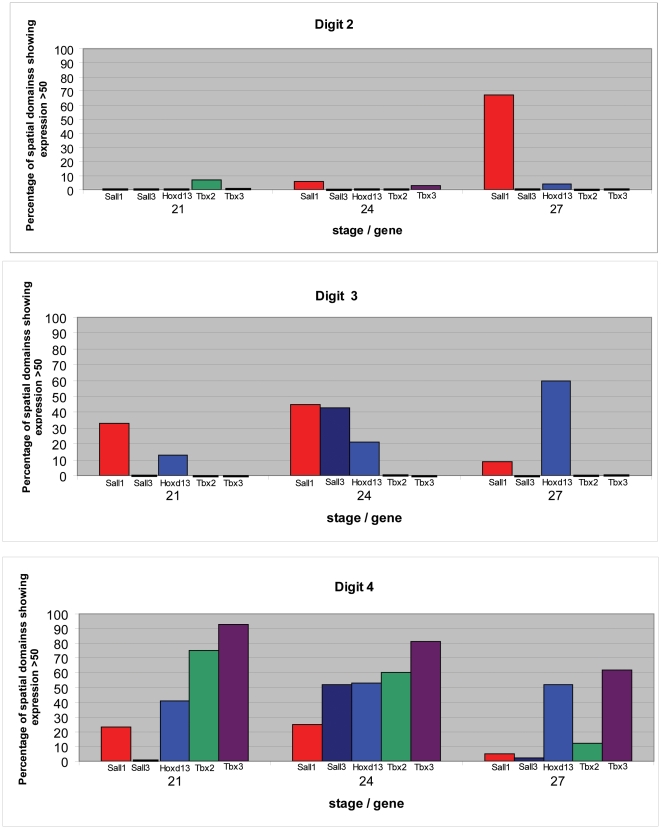
Histograms showing expression of transcription factor genes in progenitors of the three chick wing digits at three different stages. Red: *Sall1*; dark blue: *Sall3*; blue: *Hoxd13*; green: *Tbx2*; purple: *Tbx3*.

Histograms showing patterns of gene expression in progenitor cells for each of the digits at the three different stages are shown in Figure 7. For each unique digit domain, the percentage of spatial domains showing expression level >50 for each of the genes encoding the transcription factors has been calculated. Progenitors of digit 2 at stage 21 and stage 24 express none of the 5 transcription factor genes except at low levels, whereas digit 2 progenitors at stage 27, now express *Sall1* at very high levels. At all three stages, progenitors of digit 3 express *Sall1* and *Hoxd13* and, in addition, at stage 24, *Sall3*. Progenitors of digit 4 at all three stages also express *Sall1* and *Hoxd13* but in addition *Tbx2* and *Tbx3* while *Sall3* is also expressed at high levels at stage 24. While the level of *Hoxd13* expression is similar in digit 4 progenitors across all three stages, expression of the *Sall* genes is considerably reduced at stage 27.

**Figure 8:**
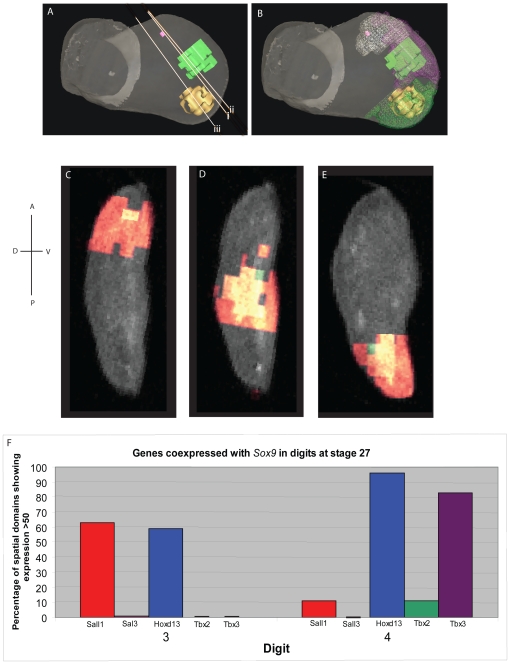
3D representations of the spatial domains expressing *Sox9* at stage 27 and histogram showing transcription factor genes expressed in them. (A) *Sox9* expression in digit 2 (pink), digit 3 (green), digit 4 (yellow). i), ii) and iii) refer to the virtual plane of section shown in C, D and E respectively. (B) *Sox9* expression domains lie within the digit progenitor domains. White mesh: digit 2; purple mesh: digit 3; green mesh: digit 4. Virtual sections shown in C, D and E. C: digit 2; D: digit 3: E: digit 4. Green: *Sox9* expression; red: other transcription factor genes; yellow: coexpression. (F) Histogram showing genes co-expressed with *Sox9* in digit 3 and digit 4 condensations.

We also co-clustered *Sox9* expression at stage 27 with expression of the transcription factor genes. As shown in Figure 8A, *Sox9* expression can be detected in a few spatial domains representing the first signs of the digit 2 condensation in addition to the two condensations for digits 3 and 4. As expected, these domains of *Sox9* expression lie within the appropriate fate map domains (Figure 8B–E). Using the tab delimited files we selected the spatial domains in which both *Sox9* and the other transcription factor genes are expressed >50 on an 8-bits greyscale. Histograms showing genes coexpressed with Sox9 are shown in Figure 8F. This computational analysis confirmed the pair-wise comparison (see above) that *Hoxd13* is expressed at high levels in the condensations for both digit 3 and digit 4, but also showed that *Sall1* is expressed at high levels in digit 3 and to a lesser extent digit 4 condensations, and *Tbx3* is expressed at high levels in digit 4 condensations.

## Discussion

We have used computer models that combine 3D expression patterns of transcription factor genes implicated in limb digit patterning and 3D fate maps to identify genes expressed in regions of the early chick wing bud that give rise to each of the three digits and then followed expression in these regions as they expand over time. The pattern of expression of some of these genes in cells with a defined fate is constant over time whereas patterns of expression other genes is much more dynamic. Thus some genes may perform the function of encoding stable positional values from early bud stages while others may play different roles at different stages including the digit condensation stage. It should be noted that this analysis has been carried out at the transcript level and protein expression patterns may differ. It should also be borne in mind that the contribution of transcription factors to digit identity can only be made explicit through functional analysis.

Progenitor cells for digit 4 express *Tbx2* and *Tbx3* from early bud stage right through to the digital plate stage, with *Tbx3* in particular being expressed at high levels in the *Sox9*-expressing condensation that will form digit 4. This would fit with a scenario in which high levels of Shh signalling in posterior regions of the early wing bud lead to expression of *Tbx2* and *Tbx3* in digit 4 progenitor cells thus encoding the cells' antero-posterior positional information; stable expression of these genes would enable this positional information to be remembered and later translated into digit identity in the forming condensations. There is also evidence that interdigital tissue controls digit identity [7] and therefore it would be interesting to map the pattern of expression of transcription factors in these regions. Functional data from over-expression of *Tbx2* and *Tbx3* suggest that *Tbx3* is involved in specifying digit III in the chick leg [27] and our recent fate map studies suggest that leg digit III is equivalent to digit 4 in the chick wing

*Hoxd13* is also expressed in progenitor cells for digits 3 and 4 at early bud stages and could again be in response to high levels of Shh signalling posteriorly. In contrast to *Tbx2* and *Tbx3*, however, although *Hoxd13* continues to be expressed at the same levels in digit 4 progenitor cells in the digital plate, it is also expressed at this stage at increased levels in digit 3 progenitors and, in addition, in digit 2 progenitors. This digital plate expression reflects initiation of the new phase of *Hoxd* gene expression suggested to be specifically involved in patterning the digit condensations [11]. In the mouse, anterior *Hoxd13* expression in the digital plate is associated with the development of digit 1 and the same is true of the most anterior digit of the chick wing [12].

Expression of *Sall1* also differs between early bud and digital plate, with *Sall1* being expressed in progenitors of both digit 3 and 4 in early bud and in progenitors of digit 2 in the digital plate. However, *Sall1* expression does not simply follow that of *Hoxd13* as there seems to be a wave of expression moving across the developing wing bud from posterior to anterior, with levels of expression not only increasing at the anterior but, at the same time, decreasing at the posterior. *Sall1* expression in the digital plate could be involved in patterning the digit 2 condensation although it should be noted that *Sall1* is also highly expressed throughout the *Sox9* expressing condensation for digit 3. In mouse double knock-outs of *Sall1* and *Sall3*, anterior digits and wrist elements are lost [29]. These defects correspond with the regions of high *Sall1* and *Sall3* expression in stage 27 chick wing buds, described here, in which *Sall1* is expressed at the anterior of the digital plate and *Sall3* just proximal to it in the region of the wrist.

Several of the genes are co-expressed in progenitors for specific digits at different stages. *Sall1* and *Hoxd13* are co-expressed in digit 3 and digit 4 progenitors at early bud stages. *Sall3* is also expressed in digit progenitors for 3 and 4 for a short period of time between early bud and digital plate stages, when it then becomes more proximal apparently excluded from the digital plate. There is evidence that *Sall1* interacts with *Hoxd13* [29] and that *Sall1* and *Sall3* interact [41]. Thus the activity of these transcription factors will vary according to whether they are present on their own or together. However our analysis does not have single cell resolution and this would be required to evaluate whether interactions between these transcription factors occur in individual cells in these domains.

Our inspiration for carrying out this analysis comes from the work on patterning of Drosophila wing venation in which expression of the Drosophila *Spalt* gene contributes to specifying vein position and identity [42] and is required for expression of *Omb* [43]. *Iroquois* is also involved in Drosophila vein specification and it has been suggested that combinations of *Sall* and *Iroquois* may specify particular veins [42]. We recently described expression patterns of *Iroquois* genes in chick wing development [44]. *Irx1* is first expressed posteriorly at stage 25 and then in digit 3 and 4 digit condensations at stage 27. Interestingly, expression of this gene then sweeps anteriorly across the digital plate later in development. It would be interesting in the future to add these expression patterns to our analysis and also look at later stages.

Visualization of expression patterns in 3D highlights dorso-ventral differences. *Hoxd13* expression is skewed dorsally in stage 21 wing buds and *Sall3* expression at stage 24, possibly reflecting a contribution by Wnt7a signalling from dorsal ectoderm to controlling expression of these genes.

These 3D models provide a framework for comparing gene expression data in the chick wing and relating these patterns to cell fate. We recently compared the expression patterns of *Tbx2*, *Tbx3*, *Sall1*, and *Hoxd13* in stage 24 chick wing buds with the expression patterns of 46 other genes, including genes identified by microarrays as being downstream of Shh signalling and genes encoding regulators of the cell cycle [45]. In this analysis, *Hoxd13* and *Sall1* were identified in the same syn-expression group consistent with this analysis. The digitized fate maps of the chick wing represent a new resource which can be used in future studies in relation to gene expression patterns.

## Materials and Methods

### Chick embryos

Fertilized White Leghorn chicken eggs were obtained from H. Stewart (Lincolnshire, UK); GFP chicken eggs were obtained from Roslin Greens, Roslin Insitute (Roslin, Midlothian, Scotland, UK).

White Leghorn chick eggs or eggs from the GFP transgenic chicken strain ([35] were incubated in a humidified incubator at 38°C for the appropriate time for the desired developmental stage as determined by Hamburger and Hamilton [31] and then windowed. On average, 50% of the eggs supplied from the GFP transgenic chicken strain are GFP-positive and these can easily be detected using a dissecting microscope with a UV light source.

### Embryo fixation for *in situ* hybridisation

Embryos were fixed in 4% (w/v) ice-cold paraformaldehyde (PFA) overnight, then put through a graded methanol series at 4°C; ending in 2×100% methanol washes and stored at −20°C until use. Eyes and forebrain were punctured with a tungsten needle to reduce trapping.

### Plasmid preparation and probe synthesis

Riboprobes were synthesised for *Sall1* and *Sall3* (from Andrea Munsterberg) *Tbx2* and *Tbx3* ( as in [22] ), *Hoxd13* ( ARK Genomics – ESTno. 414K15), *Sox9 (Elisabeth Farrell)*. A probe for GFP was made by cloning the GFP gene into a pGEM-T Easy vector and oligonucleotide primers against the GFP sequence were designed. Plasmids were grown up using standard protocols and purified using Qiagen plasmid mini kits and individual clones were sequenced to check their identity.

RNA probes were synthesised accordance with standard protocols and purified using the ProbeQuant G-50 spin column system (Amersham Biosciences).

### *In situ* hybridisation

A series of *in situ* hybridisations was performed on embryos collected at HH- stages 21, 25 and 27 for the expression of *Hoxd13*, *Tbx2*, *Tbx3*, *Sall1 and Sall3*. *In situ* hybridisation for *Sox9* was also carried out at HH-stage 27 to show early pre-cartilage condensations that have formed by this stage [39].

The patterns obtained accord with those previously described. 3–4 specimens were then selected for each gene at each time point and 3D images collected using OPT. The *in situ* hybridisation protocol used was as in [38]. For NBT/BCIP staining, we identified a particular depth of staining with the substrate that allows comparison of different probes and that is suitable for OPT scanning and subsequent mapping of gene expression patterns (see [38], supplementary materials Fig. S1). Expression patterns for *Tbx2*, *Tbx3* and *Hoxd13*, including 3D expression data can be viewed at https://www.echickatlas.org/submission/login (username GUEST, password guest).

### Fate mapping

Regions of the margin of stage 21 host chick wing buds were cut out using small iridectomy scissors and fine needles and replaced by grafts of tissue from the same region of stage 21 GFP chick wing buds. The grafts were held in place by platinum wire pins. The host embryos were then photographed with bright field and under UV light to record the initial position of the graft.

The grafted host embryo was then re-incubated at 37°C. The contributions of the grafts to the digits were examined in at 10 days of incubation. The wings were dissected and again photographed under bright field and under UV light and the fate of the GFP expressing cells recorded. Only wings that developed relatively normally e.g with the proper number of digits were scored.

In order to capture 3D images of the grafted GFP tissue as the wing developed, host wing buds were fixed for *in situ* hybridisation as above at 4 hr, 24 hr and 48 hr after grafting and *in situ* hybridisation with the GFP probe was carried out.

### Alcian green Staining

Embryos were fixed in 5% TCA (Trichloroacetic acid) overnight and then placed in 70% ethanol, 1% HCL for 2 hours. The embryos were stained in 1% Alcian Green in 70% ethanol, 1% HCL overnight and then dehydrated using 2 hour steps of successive ethanol concentrations (70%, 90% and 2×100%).

### OPT sample preparation and scanning

Reference embryos were fixed in 4% PFA/0.2% glutaraldehyde mix, which produces a stronger autofluorescent signal than PFA alone. The addition of 0.2% glutaraldehyde to the fix was not necessary for embryos that had been *in situ* hydbridized, due to the presence of glutaraldehyde in the fixative steps of that protocol. Reference embryos were stored in 100% methanol until scanning, at which point they were taken back through a methanol series to PBS and briefly to water. Embryos having undergone *in situ* hybridisation were washed 3 times for 20 minutes in PBS to remove storage fixative. In order to remove excess salts embryos were washed twice for 10 minutes in distilled water and subsequently left overnight in distilled water followed by 1 wash of 10 minutes in fresh distilled water. GFP grafted embryos were prepared for *in situ* hybridisation with the GFP probe and subsequently prepared for OPT. Alcian green stained embryos were re-hydrated back to dH2O in 2 hour sets of ethanol concentrations 90%, 70%, 50%, 25% and then in H2O for 2 hours. OPT scanning was carried out following the protocol set out in [32]. Magnification ranged from 15–23×, depending on stage (cf Fig. 2 in [38]); the prototype OPT scanner was equipped with a Leica Plan 0.5× lens. Individual scans were 268 MB. After reconstruction, the 3D Wlz objects were 220–260 MB and resolution ranged from 350×200×430 pixels to 480×450×560 pixels.

### 3D mapping

The mapping of the 3D gene expression data and the GFP fate maps to the reference models was performed using the Amira 4.1 software from Mercury Computer Systems. The data to be mapped were first roughly aligned with the reference model. Two corresponding sets of 40–100 landmarks were then set up on the isosurface for the reference embryo and the isosurface for the fluorescence/anatomical data from the scan to be mapped. The landmarks were based on prominent morphological landmarks such as the apical ectodermal ridge, the region where the limb attaches to the flank and to proportional distances along the main axes of the limb. The fluorescence/anatomical data were then warped, using a Bookstein thin plate spline (TSP) method provided by the Amira software and based on the landmark sets. Due to the nature of the TSP, the warped landmark pairs are always in perfect registration. Provided the resulting warped fluorescence/anatomical data seemed consistent with the reference limb's morphology, the same warp was then applied to the brightfield channel data. Goodness of fit of the warps was assessed by eye. The consistency of the warped data was checked by inspecting the median derived from 3–4 individual warps for each gene and by looking at the results of hierarchical clustering with TMeV, in which all replicates and the median for an individual should cluster together.

### Computational Analysis

Computational analysis of gene expression was carried out as previously described [26], [37], [45]. The programme script used to used to measure the signal intensity in the spatial domains in the reference model tabulates the data into a tab delimited (TD) file. To enable calculations, data were imported into Excel. In addition to the gene expression patterns, a unique domain for each digit was added to the dataset. Unique domains were derived from larger union domains. These union domains were calculated using the Amira arithmetic module from every domain from all grafting experiments for a specific digit at a specific stage. This gives a large domain which includes all regions of the wing bud that contributed the digit in any of the grafting experiments. The unique domains are these stage specific digit union domains masked for the union domains of the other digits, the unique domains were also calculated using the Amira arithmetic module. The unique domains represent only those regions of a specific digit's composite graft lineage, the union domain, which never overlap with the domains of the other digits' primordia grafts. Since the grafted domains showed a long extended pattern in the growing wing buds at stages 24 and 27, cropped regions were superimposed on the reference limb buds, so that only genes expressed in the distal region of the wing bud could be analysed (Figure 5A). The cropping was done manually using the Amira VolumeEdit module, the *Hoxd13* gene expression pattern was used as a guide and 2 different methods were used. A straight cropping was produced where the proximal margin of the *Hoxd13* expression pattern was used as a landmark for the placement of a plane across the P-D axis of the limb. The limb model was then cropped to retain the distal portion. The cropping was done on an orthogonal view of the dorsal surface of the developing limb with a drawn curve tracing the outline of the proximal margin of *Hoxd13* expression across the A-P axis and extending beyond the anterior - posterior margins of the limb. Everything on the distal side of the line was retained and the proximal region data discarded.

## Supporting Information

Movie S1 **showing a 3D view of unique spatial domain clusters representing regions fated to form digits in stage 21 wing bud.** Digit 2: yellow, digit 3: pink, digit 4: white.
(MPG)

Movie S2 **showing a 3D view of unique spatial domain clusters representing regions fated to form digits in stage 24 wing bud.** Digit 2: yellow, digit 3: pink, digit 4: white.
(MPG)

Movie S3 **showing a 3D view of unique spatial domain clusters representing regions fated to form digits in stage 27 wing bud.** Digit 2: yellow, digit 3: pink, digit 4: white.
(MPG)
